# Arabic translation, cultural adaptation, and validation of the Bristol Impact of Hypermobility questionnaire

**DOI:** 10.1186/s41687-023-00604-9

**Published:** 2023-06-27

**Authors:** Najla Alsiri, Meshal Alhadhoud, Asma Alhumaid, Shea Palmer

**Affiliations:** 1 Al-Razi Orthopedics and Rehabilitation Hospital, Capital governate, Kuwait; 2 Al-Adan hospital, Hadiya, Kuwait; 3 College of Biomedical & Life Sciences , Cardiff University, Cardiff, UK

**Keywords:** Hypermobility, Ehlers-Danlos syndrome, Connective tissue disorders, Laxity

## Abstract

**Background:**

The Bristol Impact of Hypermobility questionnaire (BIoH) is the first condition-specific patient reported outcome measure for people with hypermobility-related conditions. The BIoH original version is in English, which limits its use for patients who speak other languages. The study aimed to translate and culturally adapt the BIoH into Arabic and determine its concurrent validity, reliability, internal consistency and smallest detectable change.

**Methods:**

Forward-backward translation and cross-sectional designs were used. The Ethics Committee of Kuwait Ministry of Health approved the study. Spearman correlation coefficient, intraclass correlation coefficient (ICC), and Cronbach’s α were used for statistical analysis. Patients with hypermobility spectrum disorders (HSD) were included, diagnosed using the 2017 classification framework.

**Results:**

55 HSD patients were included, aged 26.0 (18.0) years old; median (IQR), and 85.5% were women. The BIoH showed very good concurrent validity when correlated with the SF-12 total and physical component scores; r = -0.743 and − 0.740, respectively (p < 0.05). Good correlation was identified between the BIoH and the SF-12 mental component score; r = -0.496 (p < 0.05). The BIoH demonstrated excellent test-retest reliability; ICC = 0.934 (0.749–0.983 95% CI) (p < 0.05), and high internal consistency (Cronbach’s α = 0.933). The smallest detectable change was 30.90 points, representing 19.8% of the mean baseline score.

**Conclusions:**

The study successfully translated the BIoH into Arabic and demonstrated high psychometric properties. The translated score can help Arabic patients with HSD in their clinical evaluation process. Future research needs to determine the responsiveness of the Arabic version and translate the BIoH to other languages.

## Key-points

• The Bristol Impact of Hypermobility questionnaire has been successfully translated into Arabic.

• This study demonstrated high psychometric properties of the translated questionnaire.

• The translated score can help Arabic patients with Hypermobility Spectrum Disorders in their clinical evaluation process.

## Introduction

Hypermobility Spectrum Disorders (HSD) are chronic connective tissue disorders characterized by symptomatic joint hypermobility etiologically related to genetic and pathologic factors [1–5]. Clinically, HSD is not uncommon reaching 30% in a musculoskeletal triage service in the United Kingdom [6]. The etiology of HSD is related to mutations in the genes encoding collagen and abnormalities in the enzymes responsible for collagen modification essential for maintaining the tissues’ mechanical strength [1, 4]. The predominant features of HSD reflect the musculoskeletal system of symptomatic joint hypermobility and instability, muscle weakness/myalgia and osteoarthrirtis [7]. However, HSD is multisystemic, including adverse effects on the cardiovascular, digestive, and autonomic nervous systems due to abnormalities in the connective tissues of these systems [2, 3, 5]. Consequently, various non-musculoskeletal features have been reported in HSD including mitral valve prolapse, irritable bowel syndrome, dysautonomia, bladder dysfunction, fatigue, sleep disturbances, functional impartment, cognitive and emotional distress, and reduced quality of life [8–11]. HSD is considered a complex and disabling condition due to its multisystemic involvement and wide range of musculoskeletal and non-musculoskeletal complaints [12]. The complexity and the disabling impact of HSD challenges healthcare professionals in robustly evaluating its impact and the effectiveness of management strategies.

Various objective examination procedures are available for evaluation and monitoring purposes for HSD such as visual analogue scale for pain, goniometry for joint range of motion, or hand-held dynamometry for hand grip strength. Despite the value of such examination tools, they reflect specific isolated impairments. Patient- reported outcome measures are vital for people with HSD to objectively evaluate the health condition and behaviors experienced directly by the patients, which is an effective way to engage the patients in measuring the quality of care as an important component in the health system [13, 14]. Patient-reported outcome measures can add more value to the clinical examination if incorporated into clinical practice in terms of quality improvement, helping in decision making, monitoring the success of management strategies and addressing the need for change [13, 14]. Burki (2021) discussed the importance of patient-reported outcome measures based: *“Patients do not really care about lung function measurements like forced vital capacity; they care about symptoms, how they feel and function in their day-to-day activities, and physicians need to care about what the patient cares about* [14].*”* The nature of HSD, as a multisystemic disorder with a potential effect on the entire body systems and the involvement of wide range of symptomatic features, requires the employment of a patient-reported outcome measure.

The Bristol Impact of Hypermobility questionnaire (BIoH) is the first condition-specific patient-reported outcome measure assessing the impact of hypermobile Ehlers Danlos Syndrome (hEDS) and joint hypermobility syndrome (JHS) [15]. The BIoH was designed to objectively evaluate people’s lives in the domains of impairment, activity, and participation [15]. This questionnaire was developed in close collaboration with patients, as it passed through three stages of a mixed method study including focus groups and interviews with patients, researchers, and clinicians, followed by think aloud interviews with patients, and finally, quantitively analysing the responses of the questionnaire [15]. It consists of 55 items to evaluate several essential components, such as average joint pain in relation to rest and activity and joint instability [15]. The three Bristol Rheumatoid Arthritis Fatigue Numerical Rating Scales were implemented in the BIoH questionnaire to assess fatigue in terms of intensity, effect, and coping [15, 16]. Additionally, the impact of the condition on various daily activities involving the upper and lower limbs were considered [15]. The BIoH showed high psychometric properties. It demonstrated excellent test-retest reliability (ICC 0.922) and went through extensive qualitative validation with patients and physiotherapists where it was evaluated positively in terms of its appropriateness, validity, acceptability, and feasibility [17, 18]. This questionnaire correlates highly against the physical component score of the SF-36 (r = 0.722) [18]. However, the original version of the BIoH questionnaire is in English, which limits its use with patients who speak other languages including Arabic. Translating the BIoH to other languages is necessary to serve patients worldwide. Twenty-two countries have the Arabic language as the official language for communication, with an estimated total population of 422 million [19]. Therefore, the aim of the present study is to translate and culturally adapt the BIoH questionnaire into Arabic and determine its concurrent validity, reliability, internal consistency and smallest detectable change.

## Methods

### Ethics and design

The Ethics Committee of Kuwait Ministry of Health approved the study in accordance with the Declaration of Helsinki (reference number: 1672/2021). Written informed consent was obtained from research participants, and privacy and confidentiality of personal information were maintained. To develop the Arabic version of the BIoH questionnaire, Forward-backward translation was followed in accordance with the guidelines of the American Association of Orthopedic Surgeons [29]. For the determination of the psychometric properties of the Arabic version of the BIoH including concurrent validity, test-retest reliability, internal consistency and smallest detectable change, a prospective cross-sectional research design was employed.

### Recruitment and eligibility criteria

Patients were recruited from the physiotherapy and orthopedic clinics of Al-Razi Orthopedics and Rehabilitation hospital, Kuwait. Physiotherapists and orthopedic surgeons were asked to screen their patients for hypermobility at the clinic visits in accordance with the 2017 HSD classification framework [20]. This framework categorizes different types of hypermobility-related disorders, through consideration of secondary musculoskeletal manifestations, and distinction from other genetic syndromes with joint hypermobility [20]. Patients who met the diagnostic criteria were invited to take part in the study via study information sheet and invitation letter. Patients who agreed to take part in the study gave their consent to pass their contact information to the principal investigator, who further confirmed the diagnosis and eligibility criteria at the examination appointment [20]. The inclusion criteria were women and men, aged ≥ 18 years old, diagnosed with HSD, able to read Arabic, with no conditions that would affect comprehension. Participants unable to comprehend Arabic or who were unwilling to participate were excluded. Participants were selected randomly for field testing from those who were diagnosed with HSD at their visits to the orthopedic and physiotherapy clinics. They were asked if they could answer the questionnaire and provide their opinions regarding the questionnaire’s clarity, relevance to the Arabic culture and ease of completion.

### Sample size justification

Following the recommendations of Gunawan, Marzilli and Aungsuroch (2021) for establishing an appropriate sample size for developing and validating a questionnaire, and the observation of variable ratio in factor analysis, a minimum sample size of 50 participants was considered sufficient for validity testing for the following reasons: (a) the current study focused on a previously developed questionnaire and not on developing a new questionnaire, (b) forward-backward translation was used to ensure accurate translation of meaning from English to Arabic, and (c) no items were removed, added or modified [21, 22]. A minimum sample size of 50 participants was also considered a feasible recruitment target from our single healthcare organization. For test-retest reliability analysis, the sample size was estimated using the intra-class correlation coefficient (ICC). A minimum sample of 10 participants was calculated, using reliability value of 0.8, α = 0.05, and power (1- ß) of 80% for repeating the measurement twice [23].

### Concurrent validity

The 12-item Short Form Health Survey (SF-12) was used to determine the concurrent validity of the Arabic version of the BIoH questionnaire. Permission to use the SF-12 was granted from John Ware Research Group of Medical Outcome Trust. The SF-12 is one of the most used generic questionnaires to evaluate health-related quality of life. It consists of 12 items focusing on physical functioning via the Physical Component Score (PCS) and mental functioning via the Mental Component Score (MCS) [24]. Several studies approved its validity, reliability, and responsiveness for the examination of general and disease-specific populations, including musculoskeletal conditions [25, 26]. It is a shorter alternative to the SF-36, involving the most predictable items for the physical and mental component scale [24]. The Arabic version of the SF-12 was previously proved as valid, reliable, and practical for measuring health-related quality of life [27, 28].

### Translation and cultural adaptation

Permission was granted from the developer of the BIoH; Professor Shea Palmer, who was also involved in this research but not specifically at the translation stage due to Arabic language barriers. In accordance with the guidelines of the American Association of Orthopedic Surgeons, this study consisted of two stages [29]. The first stage aimed to develop the Arabic version of the BIoH using forward-backward translation methods and considering cultural adaptation. The second stage aimed to determine the validity, test-retest reliability, and internal consistency of the BIoH Arabic version.

Forward translation was conducted from the original language (English) to the target language (Arabic). The Modern Standard Arabic was used for the translation, which is the Arabic countries’ official language used in formal communication. None of the Arabic Dialects were used in the translation as they differ between Arabic countries. This stage aimed to compare the translations, identify any discrepancies and discuss ambiguous and poor wording. Two independent translations were produced by two professional bilingual translators, who have the Arabic language as their mother tongue language, and were fluent in English. Challenging phrases, uncertainties, and the reasons for the final choices were documented. The two forward translators were from different backgrounds. The first translator was knowledgeable regarding the questionnaire concept and aimed to adapt the translation into a version as equivalent as possible to the original version. The second translator was not informed about the questionnaire concept to help identify any differences in interpreting the meaning of the original version when compared to the first translator. The second translator aimed to produce a version to be understood by the general population. The two translations produced were synthesized by a third translator who helped to synthesize the translations accurately and impartially without introducing any personal or cultural biases. The third translator ensured that the translations were faithful to the original content, and served as a mediator to resolve differences in translations when compared to the original version. A backward translation was then conducted to verify the overall quality of the translated questionnaire, determine any potential differences between the English and Arabic version, and ensure that they reflected similar meanings, thereby reaching a stage of conceptual equivalence. The backward translators were blind to the original version, aiming to identify any unclear wording and inconsistencies. The backward translation team involved two bilingual persons who were not informed about the concept of the questionnaire and were native Arabic speakers.

Finally, an expert committee meeting was held, including a language professional, health professional, methodologist, and the forward and backward translation teams. The expert committee mainly aimed for conceptual equivalence to consolidate all the translations between the translated version and the original version in terms of semantic, idiomatic and experimental equivalence. The expert committee developed a semi-final version for field testing. The semi-final version underwent field testing with ten patients. After answering the questionnaire each patient was interviewed to document their thoughts and responses for each item of the questionnaire. Any issues were then resolved by the expert committee and a final approved version produced after final proof reading and grammar checking.

### Psychometric properties testing

The validity of the translated questionnaire was explored by correlating the BIoH questionnaire with the SF-12 to establish its concurrent validity. For test-retest reliability and internal consistency determinations, patients were asked to answer the questionnaire at baseline then after one week. A one-week period was selected between the first and second examinations. A week was chosen as this was short enough to ensure that patient status was less likely to be altered, while ensuring that patients could still remember their original responses. Patients were asked to take part in the reliability part, when their medical history suggested that no change in health status was expected. Particularly, the eligibility criteria for the ten patients selected for reliability examination were not receiving active intervention within the one-week re-assessment period such as changes to medication, or physiotherapy, and willingness to attend after one week for re-assessment. Additionally, patients were asked at the second examination if they thought that their health status differed from the previous week. All included patients in the test-retest reliability part answered “no” to this question.

### Data collection procedures

After confirming the diagnosis and the eligibility criteria, patients were asked to complete a demographic data sheet. The Beighton Score, a measure of generalized joint hypermobility, was recorded, and the HSD classification was determined in accordance with the 2017 HSD framework including generalized, localized, peripheral or historical HSD [30]. Then the patients were asked to answer the Arabic version of the BIoH questionnaire and the Arabic version of the SF-12. Patients who were included for test-retest reliability examination were asked to re-attend after one week to answer the BIoH questionnaire to identify its reliability and internal consistency.

### Statistical analysis

Statistical Package for the Social Sciences was used for data analysis (IBM SPSS Statistics for Windows, Version 23.0. Armonk, NY, USA, IBM). Shapiro-Wilk tests were used to assess the normal distribution of the data. Most of the explored variables were significantly deviated from normality including age, weight, BMI, Beighton score, SF-12 (PCS) and SF-12 (total score) (all p < 0.05). Therefore, median and IQR were used for descriptive statistics and nonparametric tests were employed. Spearman correlation coefficient was used to assess the concurrent validity, and ICC and Cronbach’s alpha were used to assess the test-retest reliability and the internal consistency, respectively [31]. The r value was interpreted as follows; r = 0.00 to 0.20 was considered poor, 0.21–0.40 was considered fair, r = 0.41–0.60 was considered good, r = 0.61 to 0.80 was considered very good, r = 0.81 to 1.0 was considered excellent [32]. The smallest detectable change (SDC) was calculated by multiplying the standard deviation of the difference between individuals’ measurements obtained at week 1 and week 2 by 1.96 [33].

## Results

Cultural adaptation was considered during the forward-backward translation by the expert team and during field testing. However, there were no items which required cultural adaptation. In the expert committee meeting, clearer translations were discussed for the following: “given way” in questions 11 and 12, “seized up” in question 15; and “squatting” in questions 19 and 37. Conceptual equivalence was followed for the words “given way” and “seized up” to match the meaning of the original English version. The word “squatting” was directly translated to the Arabic word. Ten patients were involved in the field testing process; nine women and one man, mean (SD) age 33.5 (13.3) years, BMI of 28.0 (6.9), and Beighton score of 4.88 (1.36). Nine were categorized as GHSD and one as LHSD. The field testing showed that the Arabic version was easy to follow. No items were added, removed, or modified.

Fifty-five patients with HSD were included in the study, aged 26.0 (18.0) years old; median (IQR). Minimum age was 18 years and maximum age was 63 years. 85.5% of the included patients were women (n = 47/55). The median body mass index (BMI) was 25.2 (6.4); minimum 15.0, maximum 42.9 (Table 1). The median Beighton score was 5.0 (2.2)/9, minimum 1/9, maximum 9/9. Of the included patients, 77.2% had generalized HSD, 20.0% had localized HSD and 1.8% had peripheral HSD (Table 1). Table 1 details the demographic characteristics of the included patients and their diagnostic parameters.

**Fig. 1:**
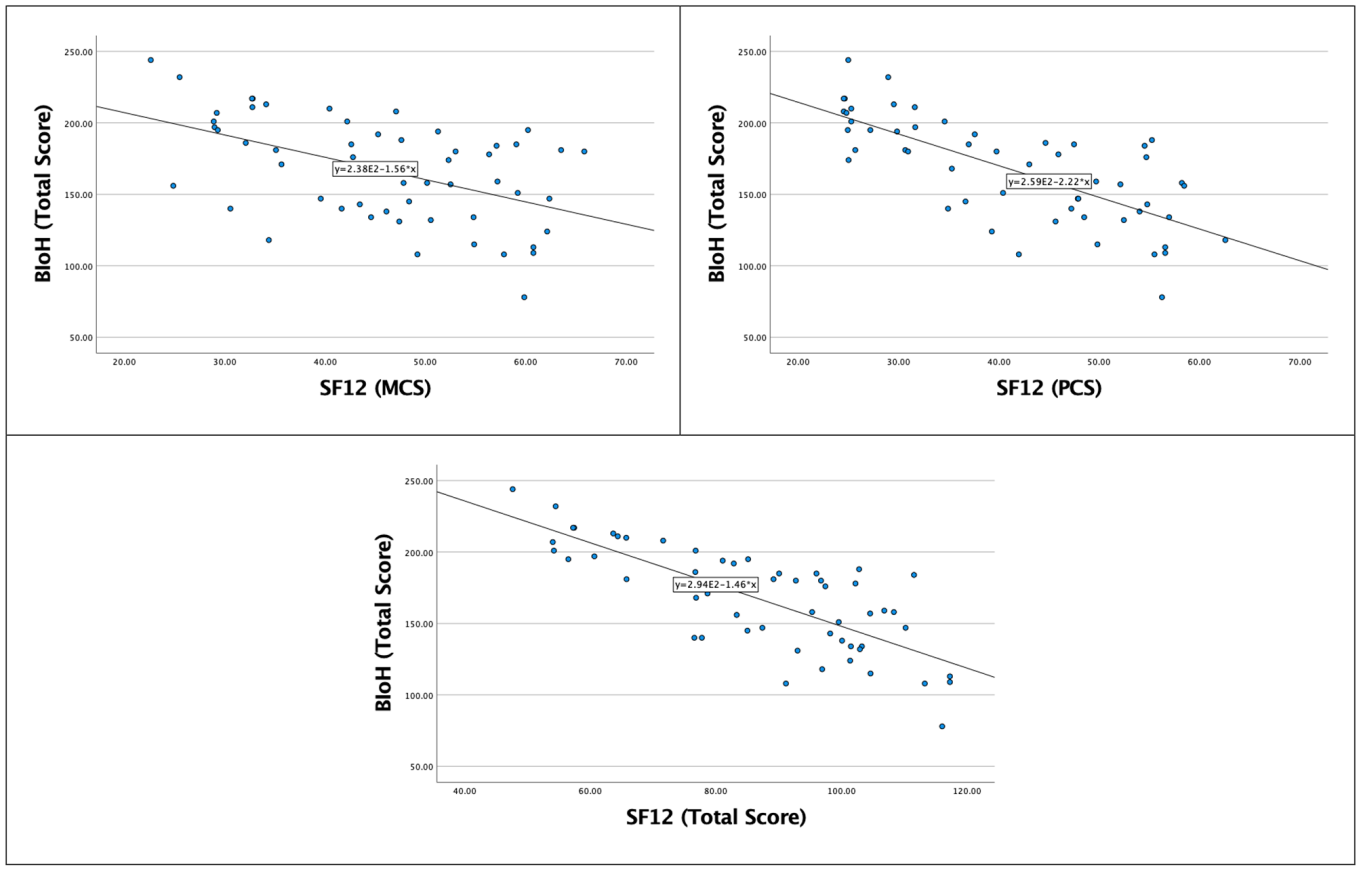
Scatterplots for the correlations between the bristol impact of hypermobility (BIoH) questionnaire and the SF-12 total score, physical component score (PCS), and mental component score (MCS)

All the included patients (n = 55) participated in the validity study of correlating the BIoH with SF-12. The BIoH demonstrated very good correlations with the total score and Physical Component Score (PCS) of the SF-12; r = -0.743 and − 0.740, respectively (both p < 0.001) (Table 2; Fig. 1). Moreover, a good correlation was identified between the BIoH and the MCS of the SF-12; r = -0.496 (p < 0.001) (Table 2; Fig. 1). Ten patients were included to examine the test-retest reliability and internal consistency of the BIoH, aged 25.0 (9.0) years, and 100% were women. Their median BMI was 24.8 (5.4), Beighton score was 5.0 (3.0)/9, and 70% were classified with generalized HSD and 30% with localized HSD. The test-retest reliability of the BIoH was high (ICC 0.934, 0.749–0.983 95% CI, p < 0.001), with high internal consistency (Cronbach’s Alpha 0.933) (Table 3). The SDC was 30.90 points, representing 19.80% of the mean baseline socre.

**Table 1:** The demographic characteristics and diagnostic parameters of the included patients with hypermobility spectrum disorder for the examination of the validity (validity group) and test-retest reliability (reliability group) of the Bristol Impact of Hypermobility questionnaire

	Validity group (n = 55)	Reliability group (n = 10)
**Age (years)**	26.0 (18.0)	25.0 (9.0)
**Sex**	85.5% women (n = 47) 14.5% men (n = 8)	100% women (n = 10)
**Height (cm)**	163.0 (12.0)	164.0 (10.0)
**Weight (Kg)**	69.0 (24.0)	63.0 (7.0)
**Body mass index**	25.2 (6.4)	24.8 (5.4)
**Beighton score**	5.0 (2.2)	5.0 (3.0)
**HSD classification**	GHSD 77.2% (n = 43) LHSD 20.0% (n = 11) PHSD 1.8% (n = 1)	GHSD 70% (n = 7) LHSD 30% (n = 3)

*Data are presented with median (interquartile range) and frequency percentage.*

*GHSD refers to generalized hypermobility spectrum disorder, LHSD refers to localized hypermobility spectrum disorder, and PHSD refers to peripheral hypermobility spectrum disorder.*

**Table 2:** The validity of the Bristol Impact of Hypermobility (BIoH) questionnaire when correlated with the SF-12; total score, physical component score (PCS) and mental component score (MCS).

Descriptive statistics Median (interquartile range)				r	p value
BIoH	174.0 (55.0)	SF-12 total score	90.1 (25.6)	-0.743	0.001*
		SF-12 (PCS)	41.9 (22.5)	-0.740	0.001*
		SF-12 (MCS)	47.0 (21.9)	-0.496	0.001*

*Correlation is significant at the 0.01 level (2-tailed)

*r = 0.00 to 0.20 poor, 0.21–0.40 fair, r = 0.41–0.60 good, r = 0.61 to 0.80 very good, r = 0.81 to 1.0 excellent* [32].

**Table 3:** The test-retest reliability of the Bristol Impact of Hypermobility Questionnaire (BIoH) when measured at baseline (BIoH1) and after one week (BIoH2) for ten patients with Hypermobility Spectrum Disorders

	Median (IQR)	ICC	(95% CI)	Cronbach’s Alpha	*P* value	*SDC*	*MD*
**BIoH1**	156.00 (42.0)	0.934	(0.749–0.983)	0.933	0.001*	30.90	-4.50 (15.77)
**BIoH2**	162.0 (62.0)						

*IQR refers to interquartile range, ICC refers to Intraclass Correlation Coefficient*

ICC: 0.00 to 0.20 poor, 0.21–0.40 fair, r = 0.41–0.60 good, r = 0.61 to 0.80 very good, r = 0.81 to 1.0 excellent [32]. *Refers to significant correlation at p < 0.05, SDC: smallest detectable change, MD: Mean Difference

## Discussion

The current study is the first study to translate the BIoH questionnaire into the Arabic language using the Modern Standard Arabic which is the formal language of communication across Arabic populations. The resultant Arabic version of the BIoH showed very good and statistically significant concurrent validity when correlated with the SF-12 total score and PCS. Moreover, excellent, and statistically significant test-retest reliability and internal consistency were highlighted for the Arabic version of the BIoH questionnaire. The SDC was determined as 19.80% (30.90 points) from the mean baseline score, which will help healthcare professionals in deciding clinically meaningful change in respect to progression of the condition or the effectiveness of the provided management.

The psychometric properties of the Arabic version of the BIoH are highly similar to properties of the original English version, suggesting that the forward backward translation was successful in transforming the meanings. Additionally, the high similarity could also be related to the fact that the original English version didn’t require cultural adaptation, so no items were removed, added or modified. The English version identified good concurrent validity when correlated with the SF-36 (PCS), with an r-value of -0.725 [15]. The present study showed similar level of concurrent validity of -0.740 when correlated with the SF-12 (PCS). The English version showed excellent test-retest reliability with an ICC of 0.923 (95% CI 0.900–0.940) and SDC of 42 points which represented a 19% change from the mean baseline score [18]. Similarly, the current study highlighted a similar level of test-retest reliability of an ICC of 0.933 (95% CI 0.749–0.983) and the SDC was 30.90 points, which is 19.90% change from the mean baseline score. However, the mean score of the BIoH determined by the present study is lower of 174.0 (55.0); median (IQR) (range 78–244), compared to the mean score determined by the previous study in which the BIoH was developed of 234 (81); median (IQR) (range 55–355) [15]. This difference in the mean score indicates that the severity of HSD is less in our sample compared to the sample of Palmer et al., (2017a), which could be related to the difference in recruitment site. Palmer et al., (2017a) recruited their patients via patient groups where patients with greater severity impact might be more likely to join support groups, while the patients in the current study were recruited via clinics [15].

The study could be limited by the small sample size of 55 participants. However, a larger sample size for score validation is only required for newly developed questionnaires [21, 22]. The English original version has previously gone through a robust validation process [15, 17, 18]. Therefore, conducting a successful forward-backward translation method during the current study ensured almost identical meaning transformation. The success of this process was evident from the similar psychometric properties identified by the current study in comparison with previous studies of the original English version. Although HSD is more common in women, it should be acknowledged that this study was conducted exclusively with women, potentially affecting the generalizability to men. The sociodemographic characteristics of the patients and their level of education could be confounders and affect generalizability, yet they were not documented and this could be considered a potential limitation.

This study has important implications for practice. It enables healthcare providers in Arabic-speaking countries to evaluate and monitor the impact of HSD on patients’ daily lives to inform the development and evaluation of management plans. The Arabic version of BIoH can also facilitate communication with Arabic-speaking patients, which can help in building trust, enhancing patient-centered care, and improving health outcomes. Additionally, the availability of the Arabic version of BIoH can raise awareness and increase knowledge about HSD among Arabic-speaking communities, and this can facilitate early diagnosis, optimize symptoms management, and minimize the overall burden of HSD. The current study is the first to translate the BIoH into Arabic and show its concurrent validity, test-retest reliability and internal consistency, which has set an important benchmark for future research. The translated score can serve as valuable tool to determine the impact of HSD on Arabic-speaking patients for both clinical and research purposes. The construct validity, responsiveness to change and other aspects of validity were not explored by the current study, which are recommended areas for future research. Future research could explore the Arabic version of BIoH on larger and more diverse populations and examine its psychometric properties. It is also recommended to translate the BIoH into more languages to serve patients with hypermobility related disorders worldwide.

## Conclusions

In conclusion, the present study successfully translated the BIoH into the Arabic language, and supported its high concurrent validity, test-retest reliability, and internal consistency and it identified the SDC. HSD is a disabling and complex condition with multisystemic involvement and symptomatic features. Therefore, the BIoH as a patient reported outcome measure is necessary as an essential component for clinical and research purposes. The current study brings forth an Arabic version of the BIoH questionnaire to serve the patients with hypermobility-related conditions who can understand Arabic, but who would not understand the English language version. Notably, there are 22 countries where the official language is Arabic with an estimated population size of 422 million. The translated questionnaire will add more value for the evaluation process and direct management strategies in an objective way and through engaging the patients in measuring the quality of care. Future research needs to determine the responsiveness of the Arabic version and translate the BIoH to other languages.

## Data Availability

Data and materials will be available upon request.

## Abbreviations

BIoH: Bristol Impact of Hypermobility questionnaire.
ICC: intraclass correlation coefficient.
hEDS: hypermobile Ehlers Danlos Syndrome.
SF-12: 12-item Short Form Health Survey.
PCS: Physical Component Score.
MCS: Mental Component Score.
IQR: Inter Quartile Range.
SMD: smallest detectable change.
GHSD: generalized hypermobility spectrum disorder.
LHSD: localized hypermobility spectrum disorder.
PHSD: peripheral hypermobility spectrum disorder.
